# The Preventive Role of the Vitamin D Endocrine System in Cervical Cancer

**DOI:** 10.3390/ijms24108665

**Published:** 2023-05-12

**Authors:** Euclides Avila, Bryan Javier Noriega-Mejía, Jocelyn González-Macías, Ulises Cortes-Hernández, Janice García-Quiroz, Rocío García-Becerra, Lorenza Díaz

**Affiliations:** 1 Departamento de Biología de la Reproducción Dr. Carlos Gual Castro, Instituto Nacional de Ciencias Médicas y Nutrición Salvador Zubirán, Av. Vasco de Quiroga No. 15, Col. Belisario Domínguez Sección XVI, Tlalpan, Ciudad de México 14080, Mexico; javiernoriegaxd@gmail.com (B.J.N.-M.); jgm9807@gmail.com (J.G.-M.); ulisescortesqfb@gmail.com (U.C.-H.); janice.garciaq@incmnsz.mx (J.G.-Q.); lorenza.diazn@incmnsz.mx (L.D.); 2 Departamento de Biología Molecular y Biotecnología, Instituto de Investigaciones Biomédicas, Universidad Nacional Autónoma de México, Av. Universidad 3000, Coyoacán, Ciudad de México 04510, Mexico; rocio.garciab@iibiomedicas.unam.mx

**Keywords:** vitamin D, human papillomavirus, cervical cancer, calcitriol, women, LSIL

## Abstract

Vitamin D along with its active metabolite calcitriol and its metabolic and signaling system, known as the vitamin D endocrine system, have been widely recognized as a pivotal regulator of calcium homeostasis in addition to non-calcemic antitumoral effects in a variety of human cancers, including cervical cancer. Several studies have found an inverse relationship between the incidence of cervical neoplasia and vitamin D levels. This narrative review updates the current evidence supporting the notion that the vitamin D endocrine system has a preventive role on cervical cancer, mainly in the early phases of the disease, acting at the level of suppressing cell proliferation, promoting apoptosis, modulating inflammatory responses, and probably favoring the clearance of human papillomavirus-dependent cervical lesions. Although an optimal vitamin D status helps in the prevention and regression of low-grade squamous intraepithelial lesions of the cervix, it appears that vitamin D alone or combined with chemotherapeutic agents has little effectivity once advanced cervical cancer is established. These observations suggest that an optimal vitamin D status might exert beneficial actions in the early phases of cervical cancer by preventing its onset and progression.

## 1. Introduction

Cervical cancer remains a major health issue affecting middle-aged women in low-income countries. Worldwide, cervical cancer is the fourth most common neoplasia in women, ranking only after breast cancer, colorectal cancer and lung cancer [1]. An estimated 604,000 new cases of cervical cancer were diagnosed globally in 2020, and about 342,000 women died of metastatic advanced cervical cancer [2]. The prevalence of invasive cervical cancer is the highest in sub-Saharan Africa, which is attributable to a combination of factors including deprivation, lower socioeconomic status and higher gender inequality, among others [3]. Fortunately, cervical cancer is highly preventable via prophylactic vaccination against the high-risk Human Papillomavirus (HPV) subtypes [4], whose chronic infection is recognized as the causal factor for the development of pre-malignant and malignant lesions of the cervix [5]. Moreover, precancerous lesions can be identified and treated using early detection screening tests based on cytologic analysis, colposcopic examination of the cervix and HPV tests [6]. These early detection programs explain why western countries have a very low incidence of invasive cervical cancer [3]. Current treatments for advanced cervical cancer have improved notably and include surgery, immunotherapy, poly (ADP-ribose) polymerase-targeted therapy, radiotherapy and standard chemotherapy [7,8].

Chronic infection with high-risk HPV subtypes is the most important risk factor for developing cervical cancer. Among the high-risk HPV subtypes, HPV16 and HPV18 account for 75% of all cases of advanced cervical cancer [5]. Of note, only a reduced percentage of chronic HPV infection cases progress to invasive cervical cancer through a very slow process, estimated to take up to 20 years or even longer. The HPV-infected cervical epithelium is first transformed to cervical intraepithelial neoplasia grade 1 (CIN1, also known as low-grade squamous intraepithelial lesion or LSIL) and later to CIN2/3 (also known as high-grade squamous intraepithelial lesion or HSIL) before developing into invasive cervical cancer [9].

In addition to high-risk chronic HPV infection, several factors contribute to cervical cancer progression and development. Some of these risk factors are related to the sexual personal history, since the chances of HPV infection increases with a younger age of sexual activity, having multiple sexual partners and full-term pregnancies, as well as young age at first full-term pregnancy [10,11,12,13]. Inflammation of the cervix caused by *Chlamydia trachomatis* and *Neisseria gonorrhoeae* infection [14] and other sexually transmitted infections as Herpes Simplex Virus type 2 infection [15] also contribute to cervical cancer progression. Chronic inflammation of the cervix by seminal prostaglandin E_2_ also increases the risk of cervical cancer [16]. Other risk factors for cervical cancer onset are immunosuppression by Human Immunodeficiency Virus infection [17], long-term use of oral contraceptives [18], smoking tobacco products [19], low educational levels [20], eating few vegetables and fruits [21,22], family history of this kind of cancer [23] and obesity [24,25]. In addition to these genetic, infectious, environmental, and lifestyle factors, there are some studies that associate vitamin D deficiency with a higher risk for cervical cancer [26,27,28,29,30,31,32]. Herein, we review the clinical, epidemiological and laboratory research evidence linking vitamin D and cervical cancer development.

## 2. The Vitamin D Endocrine System

**Figure 1:**
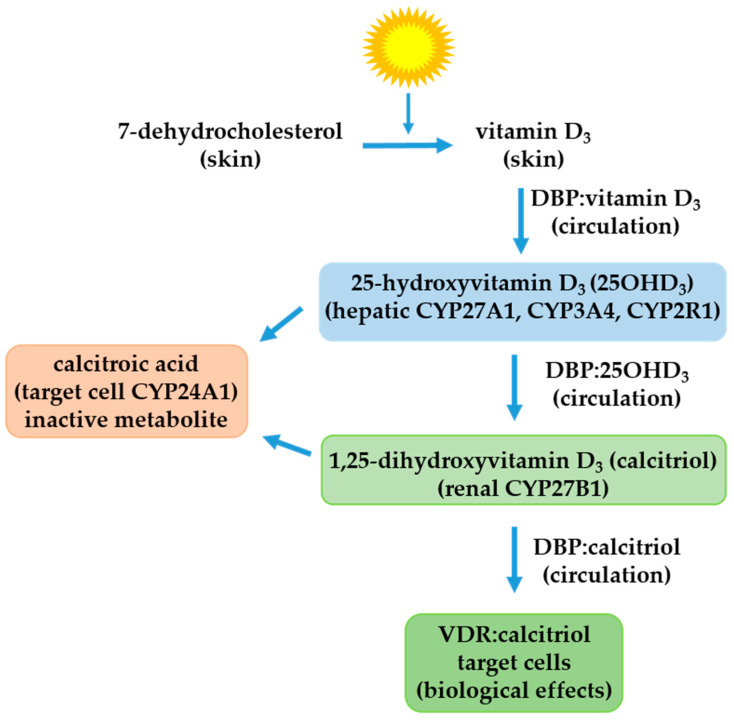
The vitamin D endocrine system. The cutaneous vitamin D_3_ formed via a sunlight-dependent process from 7-dehydrocholesterol is transported to the liver by the vitamin D-binding protein (DBP), which is 25-hydroxylated by several vitamin D_3_ 25-hydroxylases (CYP27A1, CYP3A4, and CYP2R1) to form 25-hydroxyvitamin D_3_ (25OHD_3_), which is considered the stable biomarker of vitamin D status. This metabolite is transported to the kidneys by DBP where it is hydroxylated by 25OHD_3_ 1α-hydroxylase (CYP27B1) to form 1,25-dihydroxyvitamin D_3_ or calcitriol, the hormonal form of vitamin D_3_. In vitamin D_3_ target cells containing the vitamin D receptor (VDR) and other transcriptional modulators, calcitriol regulates the transcription of mRNAs and microRNAs mediated by RNA polymerase II; otherwise, in cells expressing calcitriol-inducible 25OHD_3_ 24-hydroxylase (CYP24A1), calcitroic acid is formed, which is inactive and is excreted in bile.

The vitamin D endocrine system comprises a complex regulatory system formed by lipidic precursors, mitochondrial hydroxylases, one transporter protein, and one receptor with intrinsic activity of transcription factor, which are located in several organs including the skin, liver, kidneys, intestines, and bones (Figure 1). Considering that the major source of vitamin D in human beings is provided by vitamin D_3_ photosynthesis, in this work, we will focus only on vitamin D_3_; however, the vitamin D pool in the body refers to the sum of vitamin D_2_ (from vegetal sources) and vitamin D_3_ (from animal sources, including dairy products, oily fishes, eggs, and vitamin D_3_-fortified products).

Vitamin D_3_ is mainly formed in the skin via an ultraviolet light-dependent process from 7-dehydrocholesterol, whose 9–10 bond in the B-ring is cleaved, followed by a thermal isomerization of the molecule [33]. In this manner, fat-soluble vitamin D_3,_ also named cholecalciferol, retains the 27 atoms of carbon from its precursor 7-dehydrocholesterol, but presents an opened B-ring, which is why it is considered a secosteroid.

**Figure 2:**
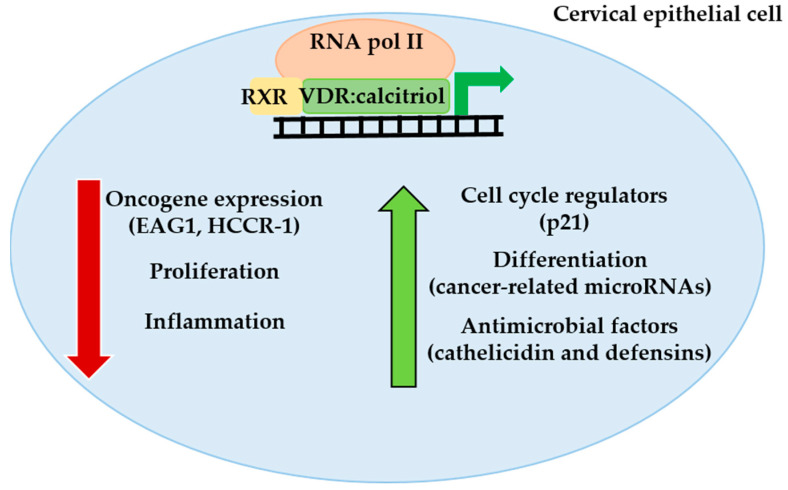
Antitumoral actions of calcitriol in cervical cancer cells. In cervical cells, calcitriol activates the vitamin D receptor (VDR), which binds with retinoid receptor X (RXR) and the heterodimer VDR-RXR is recruited to vitamin D-responsive elements in promoter regions of vitamin D-target genes, modulating the transcription mediated by RNA polymerase II. By this mechanism, calcitriol blocks the expression of some oncogenes such as the oncogenic potassium channel EAG1 or human cervical cancer oncogene HCCR-1; furthermore, calcitriol reduces proliferation and inflammation. On the other hand, calcitriol increases the expression of some critical cell cycle regulators such as p21, increases the maturation of some microRNAs with anti-cancer-related functions and promotes the expression of peptides with antiviral activity such as cathelicidin and defensins that could limit HPV infection. Together, these mechanisms support the protective role of calcitriol on cervical cancer.

Vitamin D_3_ is biologically inactive and requires two enzymatic hydroxylations to gain activity (Figure 1). The first hydroxylation of vitamin D_3_ takes place in the liver, where several proteins with 25-hydroxylase activity (CYP27A1, CYP3A4, and CYP2R1) produce 25-hydroxyvitamin D_3_ (25OHD_3_), the major circulating metabolite used to evaluate the nutritional vitamin D status [34]. Once transported to the kidneys by the vitamin D-binding protein (DBP), 25OHD_3_ undergoes a hydroxylation on position 1 by the 25OHD_3_ 1α-hydroxylase (CYP27B1) to form 1,25-dihydroxyvitamin D_3_ (calcitriol or 1,25-dihydroxycholecalciferol) [35]. Calcitriol is a potent lipidic molecule considered the hormonal form of the vitamin D endocrine system. Calcitriol activates the intracellular vitamin D receptor (VDR), and through this interaction, exerts many genomic and non-genomic biological responses not only restricted to the preservation of skeleton integrity and mineral balance of calcium and phosphate [36]. According to its classic function as a transcription factor, VDR interacts with many transcriptional modulators, including retinoid X receptor (RXR) subtypes. As a heterodimer with RXR, VDR controls the gene expression patterns of calcitriol-responsive genes by recruitment to specific DNA sequences called vitamin D-response elements (VDREs) located in the promoter regions of target genes (Figure 2) [37]. The last major component of the vitamin D endocrine system is 25OHD_3_ 24-hydroxylase (CYP24A1), the enzyme that catabolizes calcitriol as well as 25OHD_3_. This mitochondrial enzyme, highly induced by calcitriol, converts the active hormone calcitriol into inactive 24-hydroxylated products that are further oxidized to polar products that are excreted in the bile [38].

VDR expression has been detected in more than 30 different cell types [39], including the female reproductive tract [40], where uterine hypoplasia with impaired folliculogenesis was observed in VDR null mutant mice [41], highlighting the importance of this hormonal system in female fertility.

The vitamin D endocrine system action is essential for the physiology of many organs in the body, and impairment of this regulatory axis has been observed in several medical conditions such as osteoporosis, rickets, infection susceptibility, autoimmune diseases, diabetes, cardiovascular diseases, and cancer [42]. It is well established that the vitamin D endocrine system has preventive and therapeutic effects against many types of human neoplasias through a variety of mechanisms that included suppression of cell proliferation, increased apoptosis, and reduced angiogenesis, among others. These general mechanisms have been recently reviewed elsewhere [43,44]. Moreover, the roles of the vitamin D and VDR in breast, endometrial, ovarian, cervical, vulvar and vaginal neoplasias have been previously reviewed [40,45]. In this review, we updated the current knowledge of the overall vitamin D_3_ biosynthetic pathway and the new contributions made to the vitamin D_3_ actions in both in vitro and in vivo models of cervical cancer, with the aim to support the preventive role of this hormonal system in a significant global health issue in women.

Women with impaired immunity whose cervical epithelium is infected with high-risk HPVs may develop a chronic viral infection that results in genital warts, CIN1/2/3, and cervical cancer. Moreover, immune function is impaired, and inflammation markers are increased in cervical cancer patients [46]. Recently, it has been described that DBP levels are significantly decreased in the serum from cervical cancer patients compared with healthy controls, highlighting the participation of the vitamin D endocrine system in cervical cancer physiopathology [47].

## 3. The Vitamin D Endocrine System in Cervical Cancer: Preclinical Studies

It has been demonstrated that CYP27A1, CYP27B1, CYP24A1, and VDR are expressed in both normal and tumoral cervical tissue [48,49,50], which suggests local actions of this hormonal system in the cervix. Studies in HeLa cervical cancer cells demonstrated that calcitriol increased the gene expression of both CYP27A1 and CYP24A1, without affecting CYP27B1, whose mRNA was reduced by 25OHD_3_ [51]. In addition, we have reported that CYP27B1 expression and activity are detected in SiHa cervical cancer cells, which correlated with local calcitriol production from its precursor 25OHD_3_ [52]. In these cells, basal expression of CYP24A1 is detected, which is induced in the presence of calcitriol, as occurs in the vast majority of VDR-expressing cells [52]. VDR expression and activity are also detected in SiHa, and HeLa cell lines but not in C33A cells [52,53,54], where the induction of CYP24A1 gene by calcitriol is not observed [53]. It is not known why C33A lacks VDR; however, these cells might be used as negative controls to validate calcitriol-mediated effects in cervical cancer.

Although VDR is highly expressed in cervical cancer tissue compared with healthy tissue [48,49], VDR immunoreactivity is not a prognostic factor for this neoplasia, since it does not correlate with tumor stage, lymph node status, grading, histological tumor type, or the expression of the Ki-67 proliferation marker [55]. Interestingly, recent studies found that Fok1 and TaqI VDR polymorphisms are associated with a high risk of HPV16-dependent CIN2 and cervical cancer [56,57]. Fok1 and TaqI, among other VDR single-nucleotide polymorphisms, are restriction fragment length polymorphisms associated with a higher risk of gynecological neoplasias such as ovarian cancer [58]. The mechanisms by which these VDR polymorphisms promote cancer include changes in receptor affinity, DNA binding, and transcription and translation efficiency [58].

A great number of in vitro and in vivo studies have concluded that the vitamin D endocrine system has a protective role on human carcinogenesis [44]. This concept is supported by the regulatory role of vitamin D on fundamental cancer-related processes, such as cell proliferation, apoptosis, cell differentiation, angiogenesis, autophagy, inflammation, oxidative and energy metabolism, as well as immune response modulation [59]. These general mechanisms of vitamin D action have been obtained mainly from studies in different tumors, including melanoma and from colorectal, prostate and breast cancer models. In comparison, studies on the association between vitamin D and cervical cancer are scarce, and beneficial anti-cancer in vitro results have been observed only with elevated concentrations of calcitriol or its precursor 25OHD_3_, which could result in hypercalcemia in vivo. In this regard, calcitriol suppressed the proliferation of SiHa and HeLa cervical cancer cells [52,60]. In SiHa cells treated with vitamin D_3_, proliferation also is suppressed, and apoptosis is induced [61]. Thus, cell proliferation inhibition is one of the most important anti-cancer effects of the vitamin D_3_ endocrine system in cervical cancer.

Previously, we have demonstrated that the oncogenic potassium channel ether a go-go-1 (EAG1) is down-regulated by calcitriol both in HeLa and SiHa cervical cancer cell lines [52], by a mechanism involving the repressive function of a negative VDRE in the *EAG1* promoter [53]. EAG1 is a membrane protein whose normal distribution is mainly confined to the central nervous system; however, its ectopic expression is considered a carcinogenic hallmark [62]. In cervical cancer, EAG1 expression is an early marker of the disease [63], since its protein expression is detected in early CIN stages [64]. In addition, EAG1 expression is increased by estradiol in estrogen α receptor-transfected HeLa cells and also in human keratinocytes transformed by both HPV16-E6 and HPV16-E7 oncogenes [65], which highlights that EAG1 expression is up-regulated by cervical cancer-promoting factors.

Moreover, calcitriol suppresses the proliferation of HeLa cervical cells in part by a mechanism involving the down-regulation of the human cervical cancer oncogene (*HCCR-1*, LETM1 domain containing 1) [60], which encodes a mitochondrial outer membrane protein highly expressed in cervical cancer tissue [66]. Calcitriol-mediated suppression of HCCR-1 synthesis correlated with increased expression of the cyclin–dependent kinase inhibitor p21, a robust cell cycle inhibitor involved in tumorigenesis [60]. In addition, it has been demonstrated in HeLa cells that calcitriol shuts down mitochondrial homeostasis by activating the PI3K-AKT-mTOR pathway, leading to autophagy inhibition [67]. Moreover, by inducing apoptosis, vitamin D_3_ and 25OHD_3_ inhibit CaSki and SiHa cell proliferation, respectively [68,69]. These effects are the result of endogenously calcitriol produced in these cervical cells by the action of calcitriol-activating hydroxylases. Together, these evidences support the protective role of the vitamin D endocrine system on cervical cancer cell proliferation. Interestingly, despite having a high HPV16 number of copies, CaSki cells are very sensitive to the effects of antiproliferative factors. Recently, we have demonstrated that the natural compound α-mangostin inhibited the proliferation of CaSki cells, a process that was accompanied by the reduction in the expression of *EAG1* and HPV16 *E6/E7* oncogenes [70].

It has been reported that calcitriol has regulatory actions on both the expression of some components of the microRNA biogenesis machinery and the microRNA expression in VDR-positive SiHa and HeLa cells but not in C33A VDR-negative cervical cancer cells [54,71]. MicroRNAs are a class of small non-coding molecules of RNAs that globally regulate gene expression patterns. MicroRNAs are involved in a variety of fundamental cellular processes including development, apoptosis, cell differentiation and fate, cell growth, metabolic control and reproductive functions [72]. In addition, microRNA deregulation is associated with several human diseases, including cancer [72]. The canonical microRNA biogenesis pathway starts in the cell nucleus where RNA polymerase II transcribes a primary transcript named pri-microRNA, which is processed to pre-microRNA by the microprocessor complex formed by the ribonuclease Drosha, the double-stranded RNA binding protein DGCR8, and the RNA helicases DDX5, and DDX17. Once exported to the cytoplasm by the action of exportin 5/RAN-GTP, the pre-microRNA is further processed by ribonuclease Dicer to form the mature single-stranded microRNA of 18–25 nucleotides long, which is loaded onto the multiprotein RNA-induced silencing complex, leading to translational repression or mRNA degradation [72]. Among the proteins participating in microRNA biogenesis, the RNA helicase DDX5 and the ribonuclease Dicer have been induced by high concentrations of calcitriol in SiHa cervical cells through a mechanism involving the recruitment of activated VDR to inductive VDREs in their promoter regions [54,71]. These regulatory effects of calcitriol on Dicer and DDX5 were accompanied by an increased expression of several microRNAs with anti-cancer properties including microRNA-22, -29c, -4455, respectively [54]. Interestingly, tumor suppressor microRNA-22 was downregulated by HPV16 E6 in cervical cancer cells [73]. MicroRNA-29c inhibits the epithelial–mesenchymal transition in cervical cancer cells, leading to the inhibition of proliferation, invasion and metastasis [74]. The inhibition of the microRNA-4455 axis expression reduces cervical cancer growth in vitro and in vivo [75]. Thus, it appears that calcitriol-dependent increased bioavailability of DDX5 might cause the RNA substrates to be readily processed by the ribonuclease Dicer, enhancing in this way the maturation rate of microRNAs associated with anti-cancer-related functions. On the other hand, calcitriol downregulated some microRNAs in SiHa cervical cancer cells [54]; however, their role in cervical cancer remains to be investigated.

## 4. The Vitamin D Endocrine System in Cervical Cancer: Clinical Studies

The data from some clinical trials suggest that vitamin D supplementation reduces the overall cancer mortality [76]; however, other studies do not support this conclusion [77]. Regarding cervical cancer, few studies have linked the vitamin D status with the onset and progression of this pathology. Epidemiological studies showed an inverse correlation between solar irradiance and the cervical cancer incidence [27,28,78,79,80], which probably is mediated by vitamin D_3_ biosynthesis. In 2010, a Japanese case–control study including 405 patients with incidental cervical neoplasias found that cervical cancer risk was reduced by increasing vitamin D intake [26], suggesting that vitamin D plays a protective role for cervical cancer. To date, no clinical trials have been conducted that have demonstrated the expected beneficial effects of vitamin D_3_, 25OHD_3_ or calcitriol per se on women with invasive cervical cancer, but they have been developed in combination with other antineoplastic drugs, including a phase II study (NCT03192059) that enrolled cervical or endometrial cancer patients, who received a cocktail of low-dose of cyclophosphamide, aspirin, lansoprazole, vitamin D, and curcumin starting 2 weeks before radioimmunotherapy; the antitumor activity was modest but durable, and the toxicity was acceptable but not negligible [81]. A limited number of clinical trials have studied the effect of long-term vitamin D supplementation on precancerous lesions of the cervix. In this regard, an Iranian randomized, double-blind, placebo-controlled clinical trial evaluated the effects of long-term vitamin D_3_ supplementation on CIN1 regression. The study was carried out during 6 months among 58 Iranian women diagnosed with CIN1, who were randomly assigned to the placebo group (*n* = 29) or the vitamin D_3_ supplemented group (50,000 IU twice/month, *n* = 29). A higher percentage of women in the vitamin D_3_ supplemented group showed CIN1 regression when compared with the placebo group (84.6 vs. 53.8%, *p* = 0.01) [31]. Another study reported that vaginal suppositories (containing 12,500 IU of vitamin D), 3 nights *per* week during 6 weeks had good anti-inflammatory and antidysplastic effects in the CIN1 group, but no beneficial effects were seen in the CIN2 group [82]. The mechanisms explaining the protective effects of vitamin D on CIN1 regression may be related with the clearance of HPV from lesions, as has been previously suggested [29,83]. However, when another related long-term vitamin D_3_ supplementation trial was conducted in women diagnosed with more advanced cervical precancerous lesions (CIN2/3), the expected beneficial effect on recurrence was not observed [32].

Nonetheless, various studies addressing the effects of intralesional vitamin D_3_ for the treatment of HPV-induced warts have shown promising results, supporting an antiviral effect of vitamin D_3_. For example, in a single-blind randomized control trial conducted on 100 patients for one year, the intralesional vitamin D_3_ administration produced efficacious results in terms of wart clearance as well as the side effects profile [84]. Similarly, in another randomized placebo-controlled study undertaken in patients with recalcitrant extragenital warts, vitamin D_3_ treatment (600,000 IU) was effective, safe, and tolerable, with extremely low rates of recurrence [85]. The aim of this vitamin D_3_ treatment is to boost the local immune response, and the suggested mechanism of action is supposed to be related to the regulation of epidermal cells proliferation/differentiation as well as the regulation of cytokine production. In this regard, vitamin D_3_, through the action of its active metabolite calcitriol, exerts two important biological actions: an anti-inflammatory effect mediated by the inhibition of proinflammatory cytokines such as interleukin 1a, tumor necrosis factor α (TNF-α) and interleukin 6, and the induction of antimicrobial peptides synthesis [44,85,86]. The rationale behind vitamin D_3_-dependent induction of innate immunity is that, when an infectious agent activates the toll-like receptor, a concatenated sequence of events begins that include the upregulation of the VDR and CYP27B1, resulting in increased production and activity of calcitriol, which in turn transcriptionally induces antimicrobial peptides expression, such as cathelicidin [87]. Accordingly, the expression of cathelicidin after the intralesional injection of vitamin D_3_ in warts has been demonstrated, with a good clinical response [88], suggesting that the triggered immunological response definitively involves cathelicidin. Of note, not only extragenital warts have been successfully treated with vitamin D_3_, but also anogenital warts, which are also caused by HPV and commonly sexually transmitted, as observed in an adult male with condyloma acuminata, in whom, after injecting vitamin D_3_, a complete clearance of the condyloma was achieved [89]. This was also the case in anogenital warts treated topically with a vitamin D_3_ derivative [90]. Altogether, this evidence suggests a beneficial therapeutic effect of vitamin D_3_ on HPV defense, requiring further investigation.

Long-term vitamin D_3_ supplementation also had beneficial effects on glucose and lipid metabolism, and on biomarkers of oxidative stress and inflammation. These findings are of interest because chronic inflammation via several proinflammatory mediators and oxidative stress contributes to cervical carcinogenesis [16].

Therefore, the anti-inflammatory effect of calcitriol may work in a preventive manner for the development of cervical cancer, as has been previously demonstrated using a calcitriol analog in inflammation-dependent tumorigenesis of gastric cancer [91]. This anti-inflammatory effect was mediated in part by the suppression of inflammatory mediators such as cyclooxygenase-2 (COX-2) and the nuclear factor kappa-light-chain-enhancer of activated B cells (NFκB) [92]. In particular, it is known that the VDR physically interacts with IκB kinase β (IKKβ), blocking TNFα-induced IKK complex formation and NF-κB activity [92].

Accumulating evidence suggests that the vitamin D endocrine system has a protective role in developing autoimmune diseases. Accordingly, vitamin D deficiency is prevalent in patients with autoimmune diseases such as insulin-dependent diabetes mellitus, multiple sclerosis, inflammatory bowel disease, rheumatoid arthritis and systemic lupus erythematosus, where it is not known if vitamin D deficiency itself is a consequence or cause of the diseases [93,94,95]. Interestingly, a 1.5-fold higher risk of high-grade cervical dysplasia and cervical cancer has been reported in women with rheumatoid arthritis and systemic lupus erythematosus compared with women without systemic inflammatory diseases [96]. However, in a cross-sectional study in Mexico in women with systemic lupus erythematosus, the blood 25OHD_3_ concentration was not associated with cervical HPV infection [97]. Despite these results, it is well established that vitamin D is a protective agent against a broad type of infections, and vitamin D deficiency often worsens infection severity [98]. The calcitriol-dependent protective mechanisms against viral and bacterial infections involve, among others, the modulation of inflammatory immune responses and also the calcitriol-mediated synthesis of antimicrobial peptides such as cathelicidin and defensins [99]. Interestingly, α-defensin HD5 has a protective role in cervical cancer onset by blocking the HPV16 infection through capsid targeting to lysosomal degradation [100]. In this regard, more studies are needed to evaluate the possible antiviral effects of vitamin D metabolites on the HPV life cycle.

Other vitamins such as A, B9, B12, C, E, and K have also received much attention regarding cervical cancer prevention and management. For instance, women who received vitamin A supplementation had better prognoses and improved outcomes, such as clearance of HPV lesions, thereby inhibiting early cervical cancer development [101]. The vitamins B9 and B12 inhibit HPV infection and progression to CIN higher grades [102]. Additionally, it has been reported that vitamin C and E intake is inversely associated with cervical cancer risk [103]. Vitamin K has been involved in preventing the development of certain cancers, including cervical cancer [104].

However, it is noteworthy to mention that, in contrast to vitamins A, B, C, E, and K, vitamin D_3_ is not truly a vitamin [105]. Rather, it is a pro-hormone synthesized in our body in adequate quantities after sun exposure, as discussed in Section 2. In turn, 25OHD_3_ is a pre-hormone, a glandular secretory product with minimal or no significant biological activity, that is transformed in peripheral tissues into the active and potent secosteroid hormone calcitriol, whose mechanism of action compares to those of steroid hormones such as estradiol and progesterone, whom also mediate their biological effects in different target-organs via transcription factors.

Table 1 shows a summary of the clinical studies undertaken on cervical cancer, warts, and HPV-positive patients.

## 5. Conclusions

Few studies reported no beneficial effects of the vitamin D endocrine system in cervical cancer [106]. However, most studies support the concept that calcitriol and its precursors play an important preventive role in cervical cancer, as occurs in other neoplasias [107,108]. In this regard, a high intake of vitamin D is associated with a low risk of cervical cancer [26], while vitamin D deficiency is often seen in patients with CIN and invasive cervical cancer [26,29,31,32]. One of the causes of vitamin D deficiency is obesity, a risk factor for cervical cancer [25]. The low vitamin D status in obese people is related to volumetric dilution, sequestering of vitamin D metabolites in the adipose tissue and low physical activity [25]. Vitamin D deficiency also results from insufficient intake of foods rich in vitamin D and low sun exposure [25]. According to this, cervical cancer has been inversely correlated with solar irradiance, which concours with sun exposure being the most important source of vitamin D [27,28,80,109], and DBP being low in cervical cancer [47], while differential transcriptional activity by VDR polymorphisms in cervical cancer has been reported [56,57]. Moreover, calcitriol has antineoplastic activity in cervical cancer by reducing the proliferation of cell lines and the gene expression of well-known oncogenic proteins such as the potassium channel EAG1, and HCCR1 [52,60], as well as the induction of the critical cell cycle regulator p21 (Figure 2) [60]. Other potential benefits of calcitriol on cervical cancer prevention may be related to the modulation of some components of microRNA machinery biogenesis that increases the maturation of anti-cancer-related microRNAs [54,71]. Interestingly, calcitriol might have direct antiviral activity on the HPV16 life cycle, which is in line with the proposed effects of 25OHD_3_ on HPV16 replication (Figure 2) [29].

As in other neoplasias, including breast cancer, calcitriol may prevent the onset of cervical cancer; however, once established, calcitriol cannot fight advanced cervical cancer neither alone nor in combination with standard therapy, such as radiation [110].

Despite having health benefits, excess of vitamin D can produce vitamin D intoxication and hypercalcemia, whose symptoms include vomiting, frequent urination and excessive thirst. This rare but possible severe medical condition often results from uncontrolled use of vitamin D supplements [111] and, recently, from self-medication for COVID-19 infection [112].

In summary, preclinical and clinical studies reviewed here supported a protective role of the vitamin D endocrine system in preventing early HPV-dependent cervical lesions, disrupting the continuous process towards invasive cervical cancer, which suggests that an adequate vitamin D status might reduce cervical cancer incidence in women.

## Figures and Tables

**Table 1:** Clinical studies of vitamin D in cervical cancer, warts, and HPV-positive patients.

Patient Population (*n*)	Diagnostic	Principal Findings	Ref.
Japanese women Cases: 405 Controls: 11,814	Invasive cervical cancer (333 cases) or CIN3 (72 cases)	Inverse association between dietary calcium and vitamin D intake and cervical neoplasia risk.	[26]
Chinese women	The annual cancer mortality rate data were obtained from 65 rural counties included in the nationwide survey of all deaths over three years	The indices of solar UV radiation, latitude, and heat index were correlated with reduced mortality rates for cervical cancer. It suggests that vitamin D production via solar UVB reduces the risk of cervical cancer.	[27]
French women Incidence data were presented as estimates for 2000 patients	Uterine cancer	Significant positive correlations with the latitude for uterine cervix cancer, indicating that solar UVB reduces the risk of cancer through the production of vitamin D.	[28]
Turkish women Cases: 23 Controls: 62	HPV DNA-positive patients	Serum vitamin D levels were significantly lower in the study group (8085 IU/mL) than in the control group (11,472 IU/mL).	[29]
American woman Cases: 2353	Cervicovaginal HPV infection	The vitamin D level is inversely associated with the prevalence of cervicovaginal HPV infection in sexually active women.	[30]
Iranian women Cases: 29 Controls: 29	Patients with CIN1	Vitamin D_3_ administration (50,000 IU vitamin D_3_ every two weeks for six months) resulted in CIN1 regression and had beneficial effects on glucose homeostasis parameters, plasma levels of NO and MDA.	[31]
Iranian women Cases: 29 Controls: 29	Patients with CIN2/3	Vitamin D_3_ administration (50,000 IU vitamin D_3_ every two weeks for six months) had beneficial effects on the metabolic status; however, it did not affect CIN2/3 recurrence.	[32]
Chinese women Cases: 188 Controls: 188	Patients with CIN2 with HPV16-positive	Association between VDR polymorphisms (FokI and TaqI) in HPV16-positive cervical lesions and the risk of CIN2.	[56]
Thai women Cases: 204 Controls: 204	Patients with squamous cell carcinoma of the cervix	Association between VDR polymorphisms (TaqI) and cervical cancer risk.	[57]
American woman Cases: 71,209	Cervical cancer	Inverse association between UVR exposure and the incidence of cervical cancer.	[78]
American woman The cancer statistics incidence and mortality were obtained from the CDCP	Cervix uteri cancer	Association between increasing solar energy and increasing cervix uteri cancer incidence. No association between solar energy and cervix uteri cancer mortality.	[79]
Different nationalities Data from the literature	Cervical cancer	UVB irradiance significantly reduces the risk of cervical cancer.	[80]
Belgian women Cases: cervical (18) and endometrial (25) cancer	Patients with pretreated persistent/recurrent/metastatic cervical or endometrial cancer	Patients who received a drug cocktail that included vitamin D had a modest but durable antitumor activity with acceptable but not negligible toxicity.	[81]
German women Cases: 200	Women with chronic recurrent cervical infections and with cervix dysplasia (CIN 1, CIN 2)	Patients who received treatment with vaginal suppositories with 12,500 IU of vitamin D three nights per week for six weeks had good anti-inflammatory and antidysplastic effects in the CIN1 group. No beneficial effects were seen in the CIN2 group.	[82]
American women Cases: 72	Patients with prevalent high-risk HPV	A positive association between vitamin D levels and 14 high-risk HPV types persistence per 10 ng/mL increase.	[83]
Indian women and men Group A: 50, MMR vaccine Group B: 50, vitamin D_3_ treatment	Patients with single or multiple warts	The intralesional vitamin D_3_ administration produced efficacious results in terms of warts clearance as well as side effects profile.	[84]
Indian women and men Group A: 33, MMR vaccine Group B: 31, vitamin D_3_ treatment Group C: 24, saline solution	Patients with two or more recalcitrant extragenital warts of any duration at various sites of the body	The intralesional vitamin D_3_ administration (600,000 IU every two weeks, four injections, or till the complete clearance of warts) was effective, safe, and tolerable, with low recurrence rates.	[85]
Indian women and men Cases: 42	Patients with cutaneous warts	Intralesional vitamin D_3_ (600,000 IU every two weeks, four injections, or till the complete clearance of warts) was safe and effective for treating multiple cutaneous warts.	[86]
Egyptian women and men Cases: 20	Patients with verruca vulgaris	Good clinical response and expression of cathelicidin in warts after an intralesional injection of vitamin D_3_.	[88]
Indian man	Patient with condyloma acuminata	Three intralesional injections of vitamin D_3_ (600,000 IU every two weeks) resulted in complete clearance of condyloma acuminata. No recurrence was seen in a period of 6 months of follow-up.	[89]
Japanese infant	Patient with anogenital wart	Applying topical calcipotriene (vitamin D_3_ derivative) in the affected lesion twice daily for four months showed complete regression. No recurrence was seen in 6 months of follow-up.	[90]
Mexican women Cases: 67	HPV-infected women with systemic lupus erythematosus	No association between vitamin D deficiency and cervical HPV.	[97]
American women Cases: 162 Control: 12,275	Cervical cancer	Intakes of vitamin D showed no relationship with gynecological cancers.	[106]

Cervical intraepithelial neoplasia grade (CIN), malondialdehyde (MDA), nitric oxide (NO), measles, mumps, and rubella (MMR), Centers for Disease Control and Prevention (CDCP).

## Data Availability

Not applicable.
